# Transcriptomic profiling of canine decidualization and effects of antigestagens on decidualized dog uterine stromal cells

**DOI:** 10.1038/s41598-022-24790-6

**Published:** 2022-12-19

**Authors:** Miguel Tavares Pereira, Ali Kazemian, Hubert Rehrauer, Mariusz P. Kowalewski

**Affiliations:** 1 Vetsuisse Faculty, Institute of Veterinary Anatomy, University of Zurich, Zurich, Switzerland; 2 Functional Genomics Center Zurich, ETH Zurich/University of Zurich, Zurich, Switzerland; 3 Vetsuisse Faculty, Center for Clinical Studies (ZKS), University of Zurich, Zurich, Switzerland

**Keywords:** Physiology, Reproductive biology

## Abstract

Maternal-stroma derived decidual cells, the only cell population in the canine placenta expressing the nuclear progesterone (P4) receptor (PGR), are crucial for the maintenance of canine pregnancy. Decreased circulating progesterone (P4) levels, or blockage of PGR function with antigestagens, terminate canine pregnancy. As an in vitro model for canine decidualization, dog uterine stromal (DUS) cells can be decidualized in vitro with cAMP. The antigestagens aglepristone and mifepristone ablate the expression of decidualization markers in DUS cells (e.g., PGR, PRLR, IGF1 or PTGES). Here, the transcriptome profile of DUS cells was investigated to acquire deeper insights into decidualization-associated changes. Additionally, effects mediated by antigestagens (competitive PGR blockers) in decidualized cells were assessed. Decidualization led to the upregulation of 1841 differentially expressed genes (DEGs, *P* and FDR < 0.01) involved in cellular proliferation and adhesion, mesenchymal-epithelial transition, extracellular matrix organization, and vaso- and immunomodulation. The 1475 DEGs downregulated after decidualization were mostly associated with apoptosis and cell migration. In decidualized DUS cells, aglepristone modulated 1400 DEGs and mifepristone 1558 DEGs. Interestingly, around half of the identified DEGs were modulated by only one of the antigestagens. In all cases, however, PGR-blockage was mainly associated with an inversion of several decidualization-induced effects. Comparison between antigestagen-mediated effects and transcriptional changes in the canine placenta at term allowed the identification of 191 DEGs associated with diminished cell proliferation and adhesion, and vascular and immune modulation. This study emphasizes the importance of P4/PGR signaling for decidual cell function, providing new insights into the maintenance of canine pregnancy.

## Introduction

The initiation and maintenance of pregnancy fully relies on maternal recognition and coordinated interplay between fetal and maternal compartments, although the underlying mechanisms are species-specific. In most species, the primary goal of this interaction is to prevent luteolysis. In humans, the antiluteolytic signal is trophoblast-produced hCG^1,2^, while the initiation of pregnancy is signaled by embryo-derived estrogens in the pig, and by INFτ in the cow^3^. In contrast, there is no luteolytic signal in the dog, leading to prolonged activity of the corpus luteum (CL) both in pregnant and in nonpregnant animals^4,5^. Thus, the presence of an antiluteolytic factor is not required in the dog for the establishment and maintenance of pregnancy, precluding the classical maternal recognition of pregnancy^6^. Instead, maternal recognition in the dog appears to comprise a morpho-functional relationship between the uterus, the embryo, and the CL as the sole source of canine progesterone (P4), enabling initiation of pregnancy and the associated decidualization^6^. The first morphological signs of decidualization can be observed at the time of implantation, characterized by subepithelial stromal cells undergoing morpho-functional differentiation, becoming larger and rounded, and presenting an increased number of mitotic figures^7,8^.

In contrast to humans, there is no spontaneous decidualization observed in non-pregnant bitches despite high circulatory P4 levels^9^. Instead, the presence of the embryo is required for the induction of decidualization in the dog^9^. Following implantation, trophoblast invasion and placentation, stromal cells further differentiate and, within the placenta, develop towards decidual cells^8^. In species with a highly invasive hemochorial placenta, like human and rodents, the decidua plays a pivotal role in coordinating pregnancy by regulating maternal local immunity, modulating trophoblast invasion, and providing embryo nourishment^10,11^. In the shallow invasive endotheliochorial canine placenta, decidual cells, surrounded by the trophoblast, are localized in close proximity to maternal capillaries, and are the sole cellular population in the canine placenta expressing the nuclear P4 receptor (PGR)^12,13^. The expression of PGR intimately links decidual cells with the maintenance and termination of pregnancy. Late canine pregnancy is associated with a progressive decrease of P4 availability, reflecting the passive luteal regression of the CL^4^. The decreased P4/PGR signaling close to the time of parturition is associated with the activation of placental luteolytic PGF2α in the trophoblast, leading to parturition^12,14,15^. Interfering with P4/PGR signaling, e.g., by administration of antigestagens (competitive PGR blockers), like aglepristone, induces a similar signaling cascade, resulting in preterm luteolysis and/or abortion^16^. Together with aglepristone, another type II antigestagen, mifepristone, competes with P4 in binding to the PGR^16,17^. Similar to the natural ligand, i.e., P4, they cause the activation of PGR and its relocation to the nucleus^16–19^. However, unlike the P4/PGR complexes, they act as transdominant repressors, interrupting P4/PGR signaling^16,17,20^.

In a recent study, the transcriptional profiles of canine placenta derived from natural and aglepristone-induced luteolysis were compared with those of mature placenta at mid-pregnancy^21^. Luteolysis was associated with a higher transcriptional availability of genes associated with apoptosis, cholesterol transport and hypoxia, while the regulation of cell–matrix adhesion, endothelial cell function and cell cycle were affected negatively^21^. Among the top upstream regulators were, i.a., dexamethasone, TNF, TGFβ, PPARγ, as well as P4 and PGR^21^. Additionally, several genes differently expressed during luteolysis were predicted to be regulated by P4 (e.g. PTGS2/COX2, VEGF and MMPs)^21^. This further highlighted the functional importance of P4 signaling and, thereby, of decidual cells in the termination of canine pregnancy. Nevertheless, the endocrine and molecular mechanisms associated with cell–cell communication and local signaling mechanisms in the canine placenta remain to be elucidated.

The immortalized dog uterine stromal (DUS) cell line, established in our laboratory^8^, responds to stimulation with cAMP, and so serves as an in vitro model of canine decidualization^8,22,23^. During decidualization, DUS cells reflect the effects observed in vivo, becoming larger and rounder in shape, and showing increased transcriptional availability of decidualization markers, e.g. *PRLR*, *PTGES*, *IGF1* and *PGR*^7,8,23,24^. Moreover, they have an elevated expression of COL4, characteristic of epithelial cells, while retaining the expression of the mesenchymal marker vimentin (VIM), indicating their mesenchymal-epithelial transition during decidualization^8,22,23^. Conversely, treatment of decidualized DUS cells with type II antigestagens reverts the increased expression of decidualization markers and CX43, and appears to have detrimental effects on cell viability^23^.

Cumulatively, the observations so far indicate the importance of decidual cell-mediated P4/PGR signaling for the maintenance of canine pregnancy. The full nature of the underlying biological mechanisms remains, however, to be fully elucidated. Accordingly, by applying next generation sequencing (NGS, RNA-seq), we aimed to provide new insights into canine decidualization, and explore the effects evoked by antigestagen-mediated disruption of PGR signaling in decidualized canine uterine stromal cells, using our well-established model of canine decidualization, the immortalized DUS cell line. The antigestagen-mediated effects were assessed by using both aglepristone and mifepristone in decidualized DUS cells.

## Results

### Initial evaluation of sequencing results

**Figure 1:**
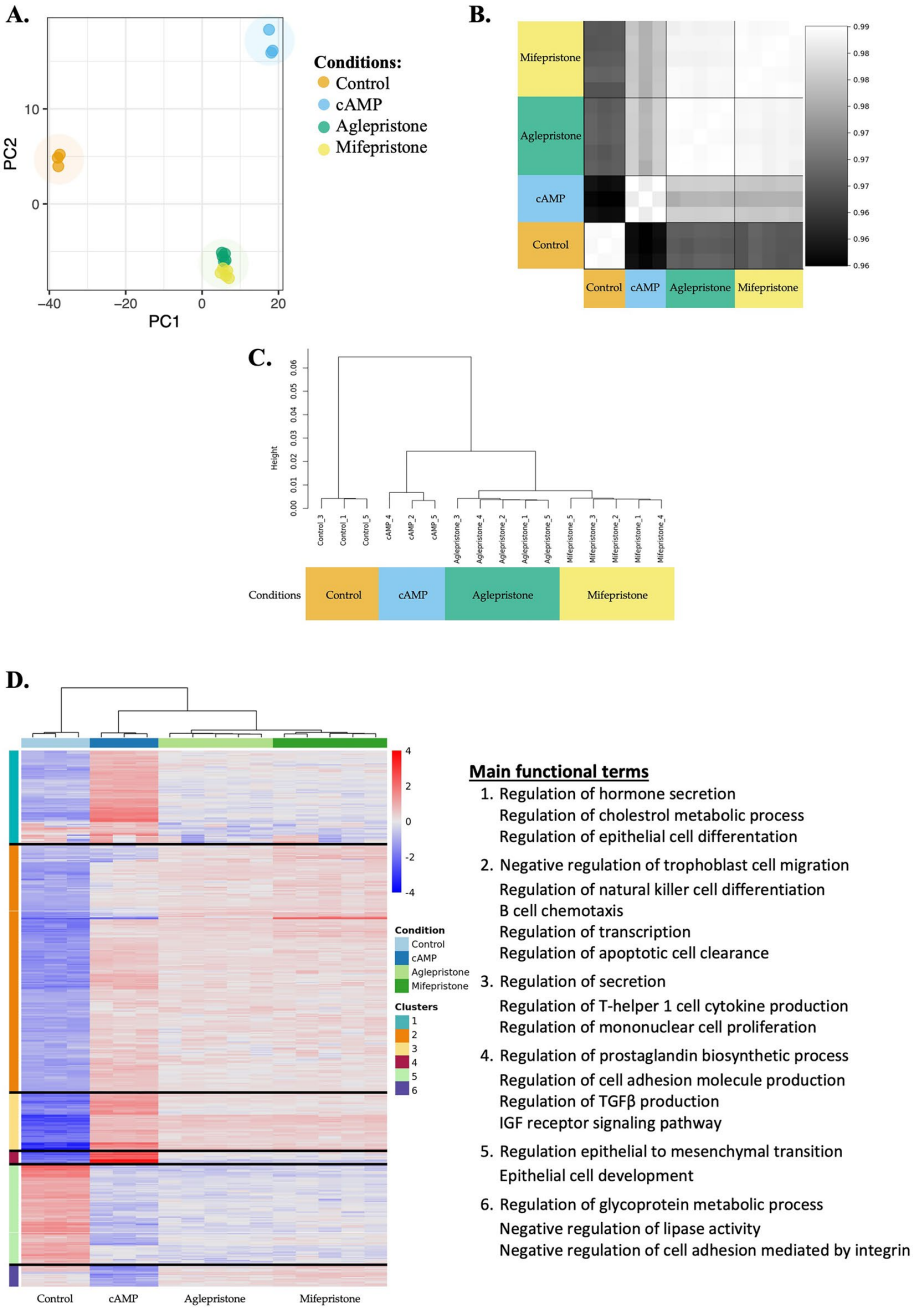
Exploratory analysis of distribution and homogeneity of samples after removal of outliers containing the 2000 genes that varied the most among all samples. (**A**) In the principal component analysis (PCA) plot of all samples, a clear separation can be observed between different treatment conditions, i.e., control, cAMP-treated and antigestagen-treated. (**B**) Samples correlation matrix shows a high homogeneity between the samples belonging to each experimental group, associated with a (**C**) clear clustering of samples from each group. (**D**) Heatmap of 2,000 genes varying the most between all samples. Colors red to blue indicate a gradient of high to low expression of each gene related to its average expression. Gene ontologies (GOs) obtained with Enrichr are presented for each cluster or genes. Clear separation of transcriptional effects was observed, in particular between control, cAMP and antigestagen-treated groups. All plots and diagrams were generated by the CountQC app available in the SUSHI framework.

The initial quality analysis of transcriptional data was performed with the CountQC app provided in the SUSHI framework, that allowed the visualization of clustering of all samples and intra-group homogeneity. Two samples from control group and two from cAMP group diverged from the other samples in the same group, in particular regarding their distribution in the principal component analysis (PCA) plot, clustering of samples and intra-group homogeneity (Supplementary Fig. 1A–C). The differences to the other samples within the respective groups could also be seen in the heatmap with the 2000 genes that varied the most among all submitted samples (Supplementary Fig. 1D). Several of the affected genes that showed high variability had low GC content (not shown), indicating that the variation observed in their expression could be associated with technical limitations. Since at least three replicates could still be obtained for the control and cAMP-treated groups, the questionable samples were excluded from further analysis. This allowed a better clustering between samples from each group, with the PCA plot showing a complete separation between control, cAMP-treated and antigestagen-treated cells (Fig. 1A). Furthermore, a higher intra-group homogeneity and clustering of samples could also be achieved (Fig. 1B,C).

A total of 32,704 genes were detected in the transcriptomic analysis, with between 12,618 and 12,747 genes being considered as expressed (minimum 10 reads) for the contrasts described below. When considering the heatmap including the 2000 genes with the highest variation among all samples, a stronger contrast between control and cAMP groups was apparent when compared with antigestagen-mediated effects (Fig. 1D). In addition, some differences between the two antigestagen groups were also visible in the heatmap (Fig. 1D), which was reflected in the separate clustering of the genes. The genes that varied the most among all samples were functionally associated with hormonal secretion, cholesterol metabolic process and epithelial cell differentiation (cluster 1); cell migration and immune-related processes (clusters 2 and 3); prostaglandin synthesis, cell adhesion, and growth factors synthesis and signaling (cluster 4); epithelial-mesenchymal transition (cluster 5); and glycoprotein metabolism, lipase activity and integrin-mediated cell adhesion (cluster 6) (Fig. 1D).

### Decidualization-associated effects:contrast “cAMP versus control”

**Figure 2:**
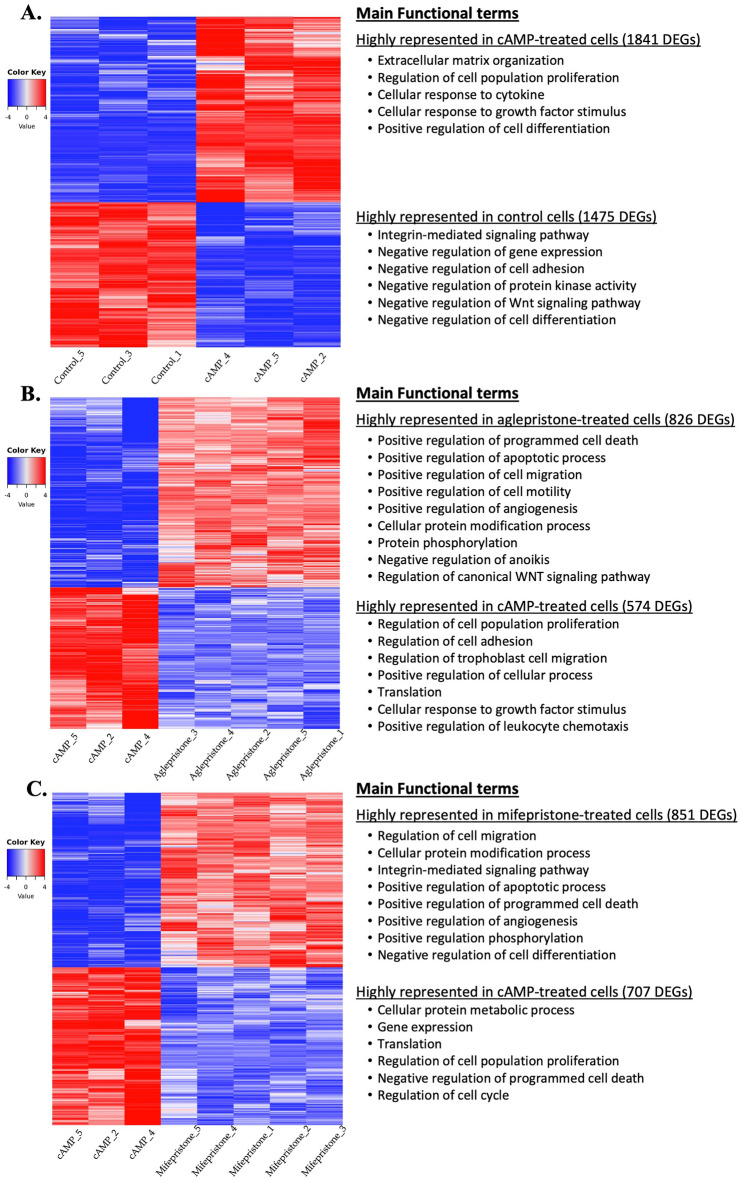
Heatmap and overrepresented gene ontologies in differentially expressed genes (DEGs) induced by different treatments (*P* < 0.01, FDR < 0.01). Gradient of expression of each gene, relative to its abundance, is represented by the colors red and blue. The main overrepresented biological process gene ontologies identified with EnrichR are listed for each contrast. The entire list of DEGs and statistical details for all contrasts are provided in the Supplementary Files 1 and 2. (**A**) Heatmap of 3316 DEGs of the contrast “cAMP versus control”. 1841 genes were more, and 1475 genes were less expressed after in vitro decidualization. (**B**) Heatmap of 1400 DEGs of the contrast “aglepristone versus cAMP”; 826 genes were more, and 574 genes were less expressed following aglepristone treatment. (**C**) Heatmap of 1558 DEGs of the contrast “mifepristone versus cAMP”; 851 genes were more, and 707 genes were less expressed following mifepristone treatment.

To investigate decidualization-induced effects, samples from the cAMP group were compared with control, i.e., non-decidualized DUS cells. This pairwise comparison was performed using the DeSeq2 package for Bioconductor. The list of differently expressed genes (DEGs) was then filtered for a *P *value and FDR lower than 0.01. A total of 3316 genes were considered as differently expressed, with 1841 DEGs being up- and 1475 DEGs being downregulated after decidualization (Fig. 2A, Supplementary file 1). Functional characterization of the DEGs was performed by identifying overrepresented gene ontologies related to biological processes with the EnrichR tool. This was later followed by the identification of enriched functional networks using the ClueGO application for Cytoscape, and prediction of overrepresented canonical pathways and top upstream regulators with IPA. Lists of up to 20 representative genes for the different functional terms and statistical details for this and other analyzed contrasts are provided in Supplementary File 2.

Genes upregulated in DUS cells during cAMP-mediated decidualization were associated with, i.a., extracellular matrix organization (*P* = 5.43 × 10^–12^), regulation of cell population proliferation (*P* = 6.21 × 10^–12^), cellular response to cytokine stimulus (*P* = 5.46 × 10^–11^) and growth factor stimulus (*P* = 1.79 × 10^–10^), and positive regulation of cell differentiation (*P* = 1.85 × 10^–6^) (Fig. 2A, Supplementary File 2). In contrast, gene ontologies identified for DEGs with decreased expression during decidualization included integrin-mediated signaling pathway (*P* = 6.39 × 10^–8^), as well as the negative regulation of gene expression (*P* = 6.97 × 10^–8^), cell adhesion (*P* = 1.94 × 10^–7^), protein kinase activity (*P* = 6.87 × 10^–6^), Wnt signaling pathway (*P* = 1.02 × 10^–5^) and cell differentiation (*P* = 3.79 × 10^–6^) (Fig. 2A, Supplementary File 2).

**Figure 3:**
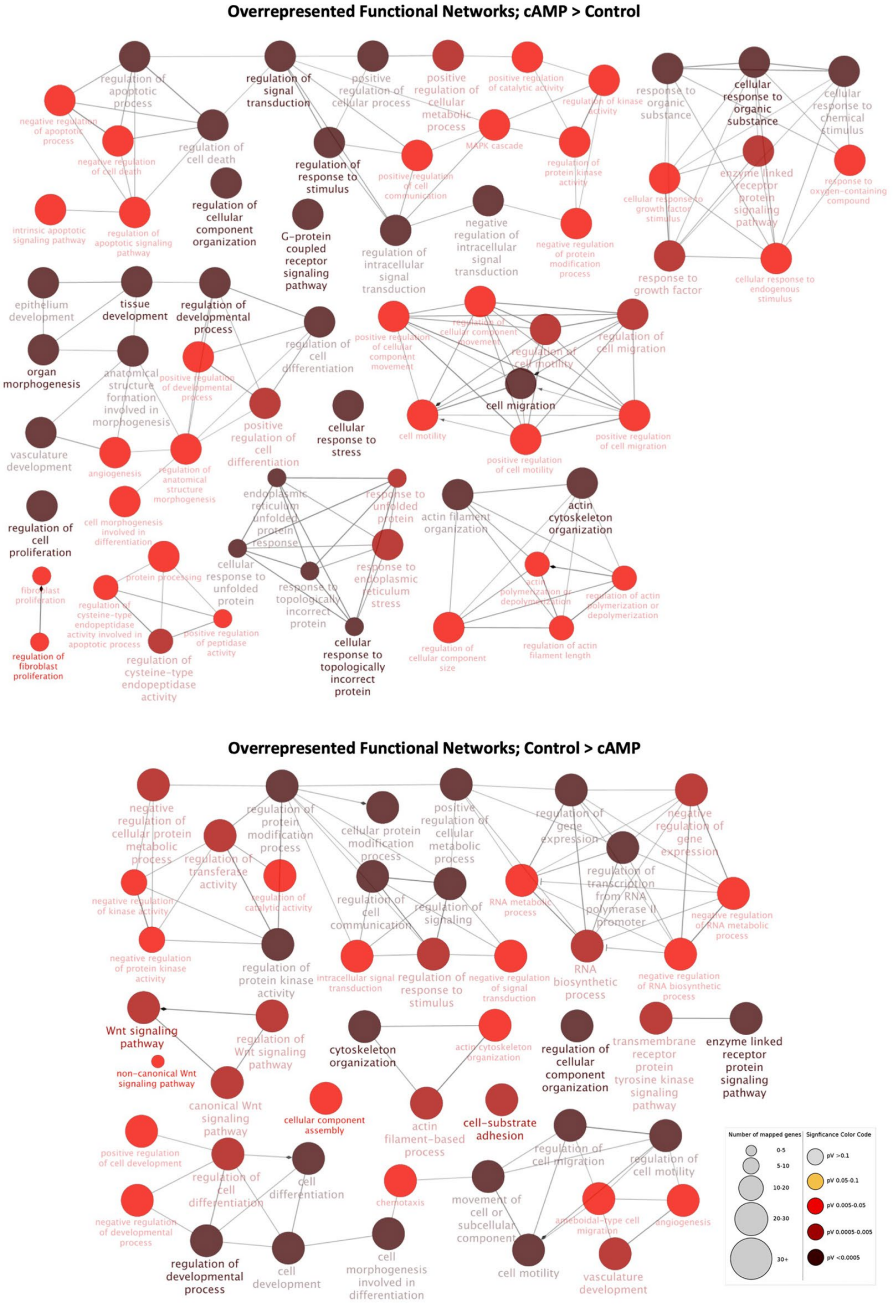
Functional networks overrepresented in the contrast “cAMP versus control”, as determined by ClueGO (Cytoscape). Redundant and non-informative terms were removed, and the resulting networks were manually rearranged. The number of mapped genes for each term is represented by the node size, whereas the level of enhancement is denoted by node color (presented in figure legend). The more highly represented functional networks for both up- and downregulated DEGs after decidualization (upper diagram) were associated with cellular migration, differentiation and cytoskeleton reorganization, angiogenesis, and regulation of kinase activity. Furthermore, networks overrepresented solely for upregulated DEGs (upper diagram) were related to cellular differentiation, response to growth factor and regulation of cell cycle and death, whereas DEGs downregulated after decidualization (lower diagram) were also associated with regulation of protein modification, gene transcription and Wnt signaling.

The ClueGO plug-in for the Cytoscape was used to group and visualize the functional networks enriched for the identified DEGs in the “cAMP versus control” contrast (Supplementary File 1). Networks more highly represented for genes upregulated after decidualization were associated with cell cycle and proliferation, response to growth factor and negative regulation of cell death (Fig. 3, Supplementary File 2). In contrast, functional networks enriched in control cells, i.e., DEGs downregulated after decidualization, were associated with Wnt signaling-related networks (Fig. 3, Supplementary File 2). Furthermore, terms associated with cell migration, differentiation and epithelization, protein kinase activity, angiogenesis and cytoskeleton reorganization were overrepresented for both up- and downregulated DEGs after decidualization (Fig. 3, Supplementary File 2).

Finally, the prediction of the most affected canonical pathways by decidualization, as well as of possible upstream regulators, was performed by uploading the full list of DEGs from this contrast to IPA. Among the canonical pathways predicted to be activated (positive z-score) after decidualization were, i.a., protein kinase A (PKA) signaling (*P* = 1.02 × 10^–10^), IL8 signaling (*P* = 3.80 × 10^–7^), p38 MAPK signaling (*P* = 8.51 × 10^–7^), cyclins and cell cycle regulation (*P* = 1.05 × 10^–5^), inhibition of MMPs (*P* = 2.24 × 10^–5^), IL1 signaling (*P* = 3.63 × 10^–4^), CXCR4 signaling (*P* = 2.19 × 10^–6^) and G-protein coupled receptor signaling (*P* = 4.27 × 10^–5^) (Supplementary File 2). Furthermore, although the activation pattern of the regulation of the epithelial-mesenchymal transition (EMT) pathway (*P* = 1.58 × 10^–11^) could not be predicted, the regulation of EMT by growth factors was predicted to be activated after decidualization (*P* = 6.31 × 10^–10^) (Supplementary File 2). In addition, cAMP treatment was associated with the deactivation of canonical pathways like integrin signaling (*P* = 1.26 × 10^–11^), IGF1 signaling (*P* = 9.33 × 10^–10^), sumoylation pathway signaling (*P* = 1.66 × 10^–10^), estrogen receptor signaling (*P* = 2.00 × 10^–9^), and prolactin signaling (*P* = 1.41 × 10^–5^) (Supplementary File 2). Finally, although predicted to be significantly affected by decidualization, no prediction on the direction of activity (undetermined z-score) could be determined for Gap junction signaling (*P* = 3.72 × 10^–6^) (Supplementary File 2).

Among the top upstream regulators predicted to affect the expression of the obtained DEGs were IL1B (*P* = 3.46 × 10^–21^), NFκB (*P* = 6.02 × 10^–13^), TNF (*P* = 7.84 × 10^–46^), FSH (*P* = 2.17 × 10^–27^), IL1 (*P* = 5.92 × 10^–11^), MAPK3 (*P* = 7.59 × 10^–6^), VEGFA (*P* = 3.00 × 10^–15^), HIF1α (*P* = 1.08 × 10^–15^), P4 (*P* = 1.71 × 10^–22^), PGE2 (*P* = 2.15 × 10^–8^), PRL (*P* = 2.03 × 10^–12^), PGR (*P* = 1.49 × 10^–6^), IGF1 (*P* = 3.88 × 10^–23^), TGFB1 (*P* = 1.14 × 10^–62^) and ESR1 (*P* = 1.22 × 10^–6^), all predicted to be activated after decidualization (Supplementary File 2).

### Antigestagen-mediated effects

#### Contrast “aglepristone versus cAMP”

To characterize antigestagen-mediated effects in decidualized cells, transcriptomic results from aglepristone- and mifepristone-treated cells were compared with the cAMP group. Treatment with aglepristone generated 1400 DEGs (*P* value and FDR < 0.01), of which 826 were more highly represented and 574 genes were negatively regulated (Fig. 2B, Supplementary File 1). Those DEGs upregulated after treatment were associated with, i.a., positive regulation of programmed cell death (*P* = 3.23 × 10^–8^), apoptotic process (*P* = 2.35 × 10^–8^), cell migration (*P* = 3.34 × 10^–11^), cell motility (*P* = 3.83 × 10^–8^), and angiogenesis (*P* = 5.51 × 10^–8^) (Fig. 2B, Supplementary File 2). Furthermore, GOs like cellular protein modification process (*P* = 3.65 × 10^–16^) and phosphorylation (*P* = 4.79 × 10^–16^), negative regulation of anoikis (*P* = 7.84 × 10^–8^), and regulation of canonical Wnt signaling pathway (*P* = 2.21 × 10^–6^) were also found for the upregulated DEGs (Fig. 2B, Supplementary File 2). The biological process ontologies enriched for the DEGs downregulated by aglepristone included: regulation of cell population proliferation (*P* = 1.92 × 10^–6^), of cell adhesion (*P* = 2.93 × 10^–5^) and of trophoblast cell migration (*P* = 5.34 × 10^–4^), positive regulation of cellular process (*P* = 2.11 × 10^–6^), translation (*P* = 5.98 × 10^–8^), cellular response to growth factor stimulus (*P* = 6.51 × 10^–4^) and positive regulation of leukocyte chemotaxis (*P* = 8.50 × 10^–4^) (Fig. 2B, Supplementary File 2).

**Figure 4:**
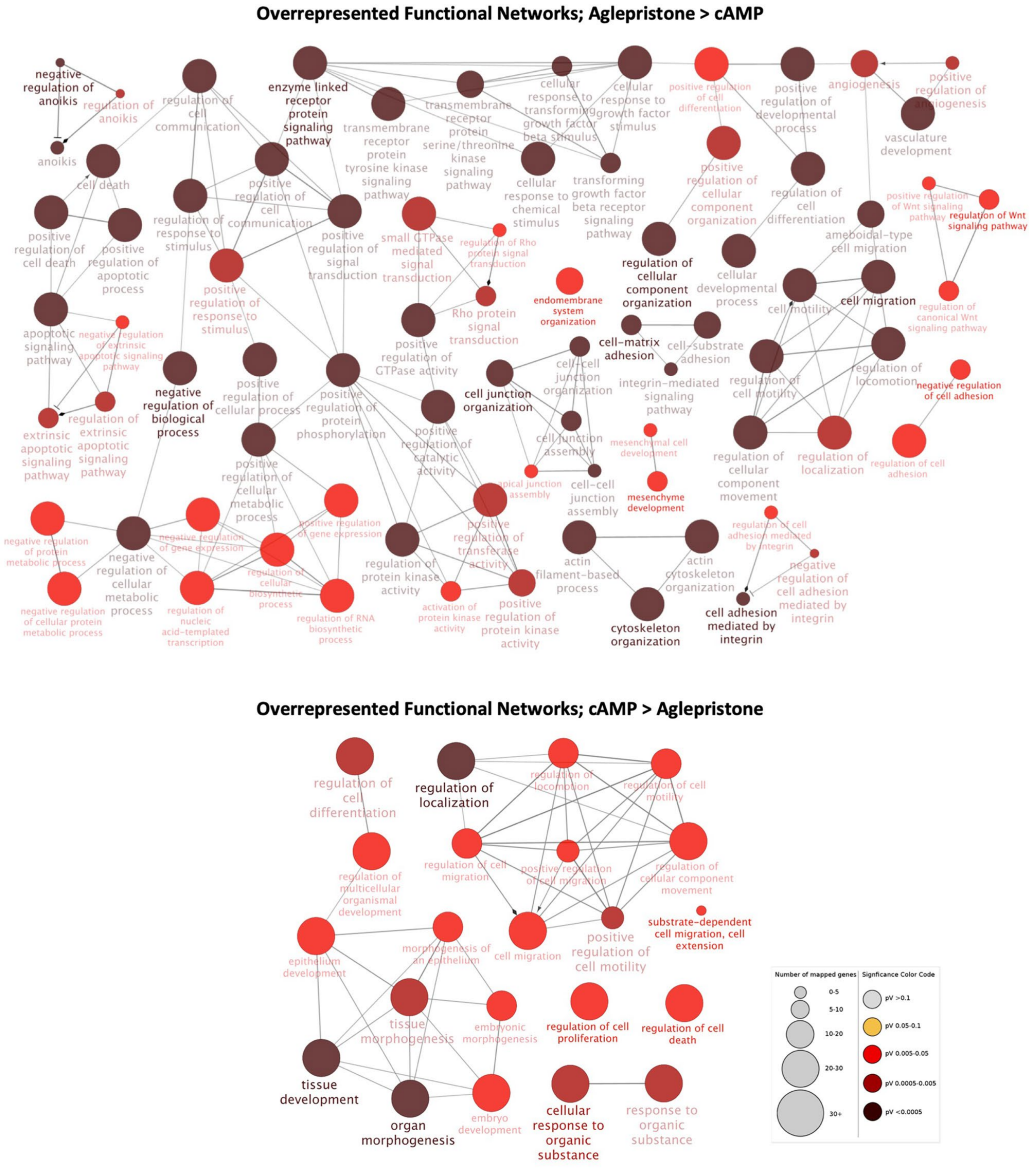
Functional networks overrepresented in the “aglepristone versus cAMP”, as determined by ClueGO (Cytoscape). Redundant and non-informative terms were removed, and the resulting networks were manually rearranged. The number of mapped genes for each term is represented by the node size, whereas the level of enhancement is denoted by node color (presented in the figure legend). Both upregulated (upper diagram) and downregulated (lower diagram) DEGs in decidualized DUS cells after aglepristone treatment were associated with the regulation of cell life cycle, cellular differentiation and migration. Functional networks solely overrepresented in upregulated DEGs were further associated with cell–cell communication and adhesion, cytoskeleton organization, anoikis and Wnt signaling, protein kinase activity, cell metabolism and angiogenesis.

The functional networks more highly represented after treatment with aglepristone were associated with cell–cell communication and adhesion, anoikis, cytoskeleton organization, protein kinase activity, angiogenesis, Wnt signaling and cell metabolic processes (Fig. 4, Supplementary File 2). Those functional networks associated with the regulation of cell proliferation, differentiation, migration and death were overrepresented both for DEGs up- and downregulated by treatment (Fig. 4, Supplementary File 2).

Among the canonical pathways predicted by the IPA software to be activated by aglepristone in decidualized DUS cells were: integrin-linked kinase (ILK) signaling (*P* = 1.58 × 10^–20^), integrin signaling (*P* = 5.01 × 10^–15^), IL-8 signaling (*P* = 1.26 × 10^–12^), IGF1 signaling (*P* = 3.98 × 10^–12^), VEGF signaling (*P* = 1.41 × 10^–9^), TGFβ signaling P = 2.04 × 10^–8^), oxytocin signaling pathway (*P* = 4.37 × 10^–8^), CXCR4 signaling (*P* = 1.45 × 10^–6^) and estrogen receptor signaling (*P* = 2.34 × 10^–6^) (Supplementary File 2). In contrast, pathways deactivated after treatment with this antigen included EIF2 signaling (*P* = 3.98 × 10^–14^), PKA signaling (*P* = 2.51 × 10^–11^), cyclins and cell cycle regulation (*P* = 3.02 × 10^–4^), and prostanoid biosynthesis (*P* = 1.74 × 10^–3^) (Supplementary File 2). Finally, the activation status of tight junction signaling (*P* = 6.03 × 10^–10^) or gap junction signaling (*P* = 3.89 × 10^–7^) could not be predicted for this set of DEGs (Supplementary File 2).

Among the upstream regulators predicted to have an increased activity associated with aglepristone treatment were TGFB1 (*P* = 6.67 × 10^–69^), beta-estradiol (*P* = 7.55 × 10^–58^), VEGF (*P* = 7.21 × 10^–24^), IGF1 (*P* = 1.46 × 10^–26^) and P4 (*P* = 8.94 × 10^–24^) (Supplementary File 2). In contrast, upstream regulators presenting a negative activation score included FSH (*P* = 1.88 × 10^–26^), CREB (*P* = 4.11 × 10^–10^), PKA (*P* = 6.98 × 10^–10^), PGE2 (*P* = 1.32 × 10^–14^), IL1β (*P* = 7.92 × 10^–26^), NFκΒ (*P* = 2.55 × 10^–7^) and PGR (*P* = 3.86 × 10^–8^) (Supplementary File 2).

#### Contrast “mifepristone versus cAMP”

Treatment of decidualized DUS cells with mifepristone generated 1558 DEGs (*P* value and FDR < 0.01; Fig. 2C, Supplementary File 1). Of those, 851 DEGs were upregulated and 707 were downregulated. The DEGs upregulated by mifepristone were associated with ontologies such as regulation of cell migration (*P* = 8.07 × 10^–18^), cellular protein modification process (*P* = 2.11 × 10^–13^), integrin-mediated signaling pathway (*P* = 3.01 × 10^–12^), positive regulation of apoptotic process (*P* = 4.81 × 10^–9^), programmed cell death (*P* = 6.03 × 10^–9^), angiogenesis (*P* = 3.27 × 10^–9^), and of phosphorylation (*P* = 7.89 × 10^–10^), and negative regulation of cell differentiation (*P* = 1.07 × 10^–8^) (Fig. 2C, Supplementary File 2). In contrast, downregulated DEGs were associated, i.a., with cellular protein metabolic process (*P* = 1.44 × 10^–18^), gene expression (*P* = 1.19 × 10^–13^) and translation (*P* = 1.55 × 10^–16^), regulation of cell population proliferation (*P* = 5.12 × 10^–6^), negative regulation of programmed cell death (*P* = 9.69 × 10^–5^) and regulation of cell cycle (*P* = 1.47 × 10^–4^) (Fig. 2C, Supplementary File 2).

**Figure 5:**
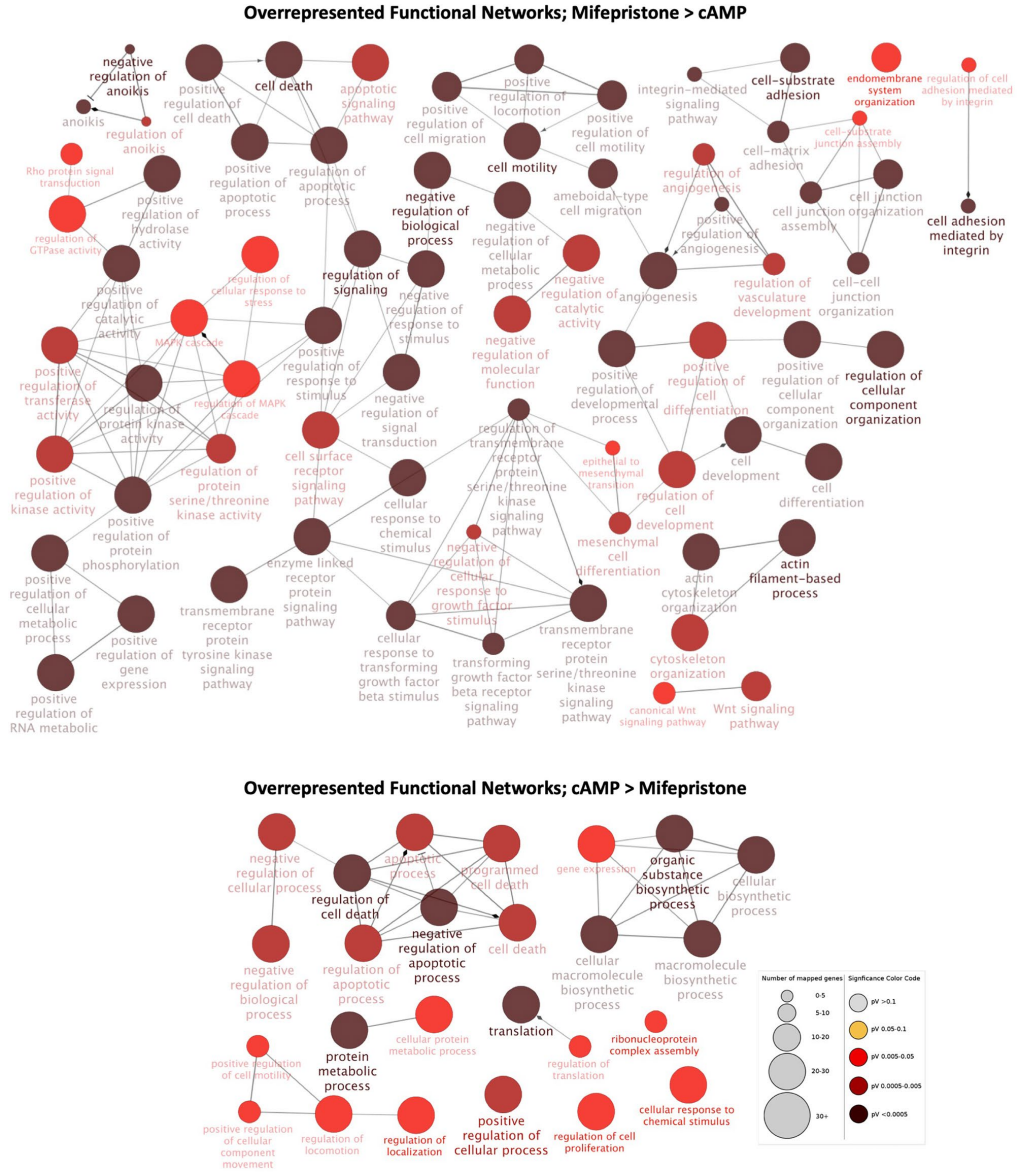
Functional networks overrepresented in the contrast “mifepristone versus cAMP” as determined by ClueGO (Cytoscape). Redundant and non-informative terms were removed, and the resulting networks were manually rearranged. The number of mapped genes for each term is represented by the node size, whereas the level of enhancement is denoted by node color (presented in the figure legend). The overrepresented functional networks for the DEGs upregulated in decidualized DUS cells by treatment with mifepristone (upper diagram) were mainly related to the apoptotic process, anoikis and Wnt signaling, cell motility and adhesion, epithelial mesenchymal transition, response to growth factors, protein kinase activity and angiogenesis. Networks overrepresented in the DEGs downregulated by mifepristone (lower diagram) were, similar to upregulated DEG, associated with cell death and mobility, while also being related to translation and cellular metabolism.

Functional networks enriched for upregulated DEGs after mifepristone treatment were associated with the regulation of apoptosis and cell death, cell motility and (integrin mediated) adhesion, angiogenesis, regulation of mesenchymal differentiation, response to stimulus, protein kinase activity and Wnt signaling (Fig. 5, Supplementary File 2). As for downregulated DEGs, besides sharing functional networks associated with cell death and mobility, translation and cellular metabolism associated terms were also enriched (Fig. 5, Supplementary File 2).

The treatment of decidualized DUS cells with mifepristone was associated with a predicted activation of canonical pathways such as ILK signaling (*P* = 6.31 × 10^–23^), integrin signaling (*P* = 3.98 × 10^–15^), actin cytoskeleton signaling (*P* = 1.58 × 10^–11^), IL-8 signaling (*P* = 5.01 × 10^–11^), IGF1 signaling (*P* = 8.13 × 10^–9^), VEGF signaling (*P* = 2.82 × 10^–7^), CXCR4 signaling (*P* = 4.17 × 10^–6^) and estrogen receptor signaling (*P* = 4.68 × 10^–5^) (Supplementary File 2). In contrast, pathways like EIF2 signaling (*P* = 6.31 × 10^–28^), PKA signaling (*P* = 3.80 × 10^–8^), oxytocin signaling (*P* = 7.24 × 10^–5^), prostanoid biosynthesis (*P* = 2.69 × 10^–3^) or IL-1 signaling (*P* = 8.91 × 10^–3^) were predicted to be deactivated by the IPA software in response to mifepristone, whereas no activation pattern could be predicted for tight junction signaling (*P* = 4.27 × 10^–7^) or glucocorticoid receptor signaling (*P* = 4.17 × 10^–4^) (Supplementary File 2).

Finally, TGFB1 (*P* = 1.29 × 10^–63^), ESR1 (*P* = 1.12 × 10^–5^), VEGF (*P* = 6.98 × 10^–10^), IGF1 (*P* = 3.92 × 10^–23^), P4 (*P* = 5.44 × 10^–23^), or caspase (*P* = 1.03 × 10^–2^) were among the predicted activated upstream regulators for this contrast (Supplementary File 2). On the other hand, upstream regulators predicted to have a negative activation score included CREB (*P* = 2.13 × 10^–9^), PKA (*P* = 1.83 × 10^–6^), FSH (*P* = 6.24 × 10^–25^), IL1 (*P* = 1.57 × 10^–10^) and PGR (*P* = 9.52 × 10^–10^) (Supplementary File 2).

### Intersection of DEGs modulated under different treatment conditions, and comparison with prepartum luteolysis (in vivo)

**Figure 6:**
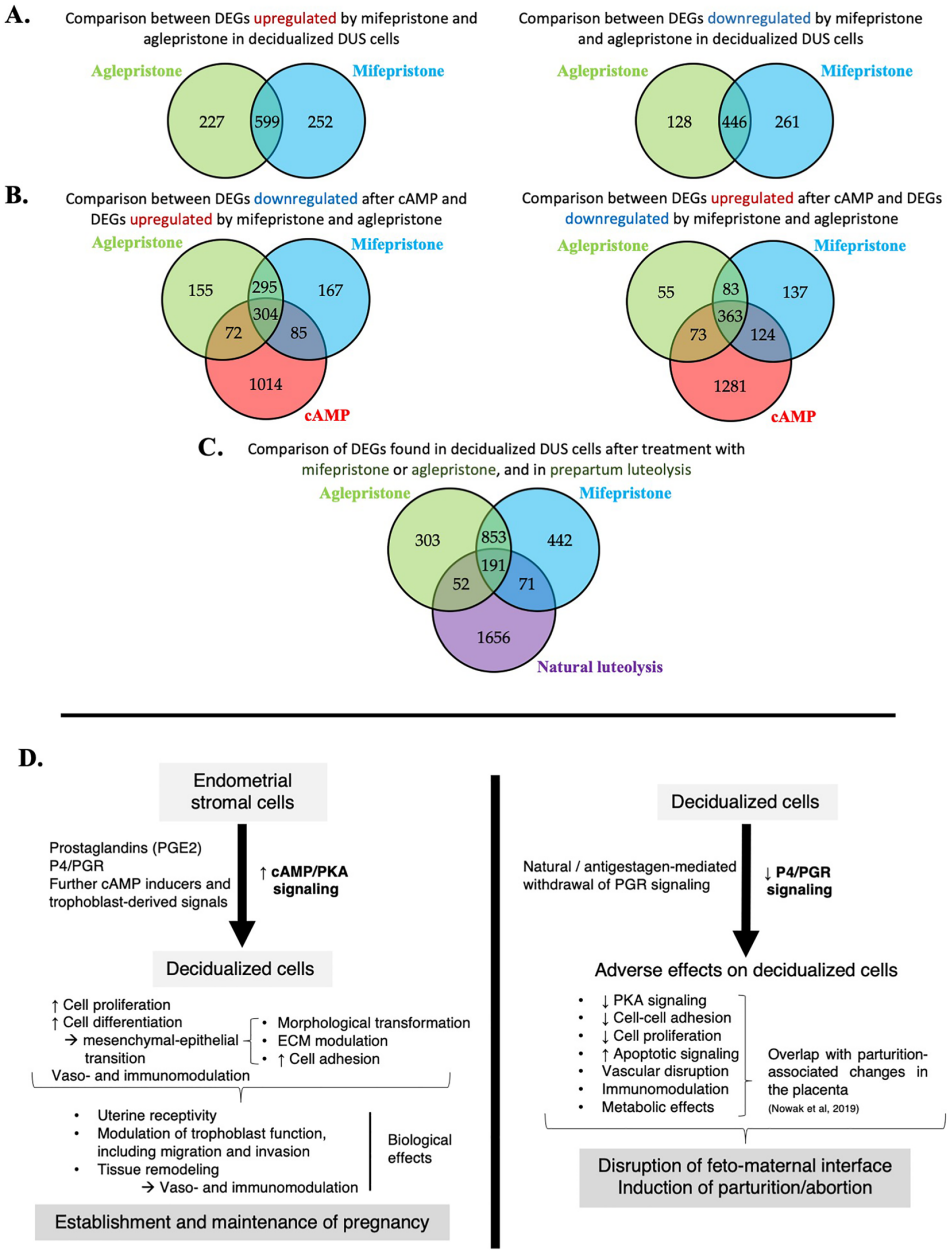
Venn diagrams showing the distribution and overlap of DEGs between the different analyzed contrasts, and schematic representation of decidualization-associated changes in dog uterine stromal cells and antigestagen-induced effects in decidualized cells. (**A**) Intersection between list of DEGs from the contrasts “aglepristone versus cAMP” and “mifepristone versus cAMP”. (**B**) The intersection between DEGs downregulated in the contrast “cAMP versus control” and upregulated/downregulated in the contrasts “aglepristone versus cAMP” and “mifepristone versus cAMP”. (**C**) Intersection between the list of both up- and downregulated DEGs from “aglepristone versus cAMP” and “mifepristone versus cAMP”, and genes differentially expressed in the canine placenta at the time of prepartum luteolysis, when compared with mid-term stage [contrast “natural luteolysis versus mid-gestation”, *P* < 0.01, FDR < 0.1, presented in^21^]. Lists of DEGs (*P* < 0.01, FDR < 0.01, unless stated otherwise) from respective comparisons were used, with the complete list of genes from each intersection being provided in Supplementary File 3. (** D**) The main findings of the present work are summarized, taking into consideration previous descriptions^8,21–24^. A detailed explanation is provided in the text.

To evaluate the similarities between different treatment conditions, the intersection of DEGs from different contrasts were visualized with Venn diagrams (Fig. 6). The complete list of genes at each intersection, as well as details regarding functional terms identified, are described in Supplementary Files 3 and 4. The transcriptional effects induced by both antigestagens on decidualized DUS cells were compared using the DEGs obtained for the contrasts “aglepristone versus cAMP” and “mifepristone versus cAMP”. About half of all identified DEGs [55.6% of upregulated (599 DEGs) and 53.4% of downregulated (446 DEGs)] were commonly modulated by both antigestagens (Fig. 6A, Supplementary File 3). The DEGs upregulated by both antigestagens were involved, i.a., in the regulation of cell migration (*P* = 2.19 × 10^–17^), regulation of apoptotic process (*P* = 2.55 × 10^–11^), integrin-mediated signaling pathway (*P* = 5.93 × 10^–11^), regulation of cell–matrix adhesion (*P* = 6.00 × 10^–9^), and angiogenesis (*P* = 1.07 × 10^–8^) (Supplementary File 4). On the other hand, the gene ontologies enriched for the DEGs concomitantly downregulated by aglepristone and mifepristone included translation (*P* = 2.89 × 10^–9^), regulation of cell population proliferation (*P* = 7.65 × 10^–8^), cellular response to cytokine stimulus (*P* = 4.74 × 10^–7^), and regulation of Wnt signaling pathways (*P* = 1.91 × 10^–4^) and of trophoblast cell migration (*P* = 2.04 × 10^–4^) (Supplementary File 4).

Next, the effects of antigestagens on genes modulated (up- and downregulated) during in vitro decidualization were assessed by comparing DEGs generated by antigestagen treatment of decidualized DUS cells. DEGs upregulated in the contrast “cAMP versus control” (i.e. characteristic of decidualization) were compared with DEGs found to be downregulated in the contrasts “aglepristone versus cAMP” and “mifepristone versus cAMP”, whereas the DEGs downregulated after decidualization were compared with those upregulated by antigestagens. Of the 1475 DEGs downregulated during in vitro decidualization, 304 (20.6%) were concomitantly upregulated by antigestagens (Fig. 6B, Supplementary File 3). These overlapping DEGs were associated with gene ontologies like regulation of cell migration (*P* = 1.83 × 10^–13^), regulation of cell adhesion mediated by integrins (*P* = 6.62 × 10^–7^), positive regulation of apoptotic process (*P* = 5.85 × 10^–6^), regulation of Wnt signaling pathway (*P* = 8.23 × 10^–6^), cell–cell junction organization (*P* = 4.27 × 10^–6^), positive regulation of angiogenesis (*P* = 7.07 × 10^–5^) and of epithelial-mesenchymal transition (*P* = 2.00 × 10^–5^) (Supplementary File 4).

Conversely, of the 1841 DEGs upregulated by cAMP-mediated decidualization, 363 (19.7%) were concomitantly downregulated by both antigestagens (Fig. 6B, Supplementary File 3). Biological process GOs enriched for this intersection included the regulation of cell population proliferation (*P* = 6.22 × 10^–8^), cellular response to cytokine stimulus (*P* = 2.52 × 10^–7^) and to growth factor stimulus (*P* = 3.27 × 10^–5^), regulation of cell adhesion (*P* = 3.17 × 10^–5^) and of trophoblast migration (*P* = 9.25 × 10^–5^), and positive regulation of protein kinase activity (*P* = 5.21 × 10^–4^) (Supplementary File 4).

Finally, the effects evoked in decidualized DUS cells by antigestagens coincided with transcriptional changes observed in the canine placenta during prepartum luteolysis. The previously published dataset GSE126031, more specifically the contrast “prepartum luteolysis versus mid-gestation”^21^, was involved in this comparison. The latter contrast allowed identification of 1973 DEGs (*P* < 0.01, FDR < 0.1) in the canine placenta associated with the onset of parturition^21^. A total of 191 DEGs associated with prepartum luteolysis were commonly affected by mifepristone and aglepristone in decidualized DUS cells (Fig. 6C, Supplementary File 3). These genes were mainly associated with biological processes like regulation of cell migration (*P* = 9.33 × 10^–6^), negative regulation of transmembrane receptor protein serine/threonine kinase signaling pathway (*P* = 8.03 × 10^–5^), negative regulation of cellular response to growth factor stimulus (*P* = 1.17 × 10^–4^), regulation of cell population proliferation (*P* = 1.38 × 10^–4^), angiogenesis (*P* = 1.50 × 10^–4^) and of apoptotic process (*P* = 2.80 × 10^–4^), and regulation of the epithelial to mesenchymal transition (*P* = 6.79 × 10^–4^ (Supplementary File 4). The lists of respective genes are presented in Supplementary File 3.

### Expression of candidate genes

The mRNA availability of genes selected from DEGs obtained for different contrasts was evaluated by semi-quantitative real-time TaqMan PCR to further validate the NGS results. Included were representative genes for different functional groups identified as affected by decidualization and/or antigestagens: growth factors (*AREG*, *HGF*, *PAPPA2*, *ANXA2*), immune factors (*CXCL10*, *IL1R1*, *TGFβR2*, *NFKBIA, PTGS2*, *LIFR*), factors related to activities of kinases (*CDKN1A*, *AKAP12*), cell adhesion factors (*SELP*, *ITGA9*, *LAMA4*, *CDH1*), vascular regulators (*EDNRB, THBS2*, *VEGFR1* and *ANGPT4*), glucose and lipid transporter (*SLC2A1*, *LDLR*), and hypoxia related factor *HIF1α*. In general, the relative expression patterns of selected candidate genes followed the results obtained from RNA-Seq. In vitro decidualization achieved by treatment of DUS cells with cAMP was associated with a significantly increased expression of *AREG*, *HGF*, *CXCL10*, *IL1R1*, *TGFR2*, *PTGS2*, *CDKN1A*, *AKAP12*, *SELP*, *LAMA4*, *EDNRB, THBS2* and *SLC2A1*, when compared with non-decidualized control DUS cells (*P* < 0.001, Table 1). Conversely, the expression of *LIFR*, *ITGA9*, *CDH1*, *VEGFR1* and *ANGPT4* (*P* < 0.001), and of *NFKBIA* (*P* < 0.01) decreased during decidualization process (Table 1). In contrast, no significant differences between control and cAMP groups were found for *LDLR* (*P* > 0.05, Table 1). Furthermore, although the mRNA availability of *PAPPA2* was below detection limits in control samples, it could be detected after decidualization (Table 1). For this reason, control group was removed from statistical analysis for *PAPPA2*.

**Table 1:** Relative gene expression of selected candidate genes affected by different treatment conditions.

Gene symbol	Control (RGE ± SD)	0.5 mM cAMP (RGE ± SD)	1 μM Aglepristone (RGE ± SD)	1 μM Mifepristone (RGE ± SD)	Anova	Tukey–Kramer test	
*AREG*	1 ± 0.00	773.36 ± 361.64	99.78 ± 52.93	106.42 ± 41.40	*P* < 0.0001	Control versus cAMP cAMP versus Aglepristone cAMP versus Mifepristone	*P* < 0.001 *P* < 0.001 *P* < 0.001
*HGF*	1 ± 0.00	55.11 ± 16.89	17.73 ± 6.34	14.25 ± 2.64	*P* < 0.0001	Control versus cAMP cAMP versus Aglepristone cAMP versus Mifepristone	*P* < 0.001 *P* < 0.001 *P* < 0.001
*PAPPA2*	Below detection limits	1 ± 0.00	0.37 ± 0.09	0.30 ± 0.11	*P* < 0.0001	cAMP versus Aglepristone cAMP versus Mifepristone	*P* < 0.001 *P* < 0.001
*ANXA2*	1 ± 0.00	0.98 ± 0.32	1.07 ± 0.25	0.90 ± 0.14	*P* = 0.431	–	
*CXCL10*	1 ± 0.00	242.81 ± 137.96	20.41 ± 5.97	15.09 ± 7.55	*P* < 0.0001	Control versus cAMP cAMP versus Aglepristone cAMP versus Mifepristone	*P* < 0.001 *P* < 0.001 *P* < 0.001
*IL1R1*	1 ± 0.00	12.36 ± 6.62	6.95 ± 1.48	4.28 ± 1.36	*P* < 0.0001	Control versus cAMP cAMP versus Aglepristone cAMP versus Mifepristone	*P* < 0.001 *P* < 0.05 *P* < 0.001
*TGFBR2*	1 ± 0.00	2.50 ± 0.83	1.48 ± 0.39	1.66 ± 0.23	*P* < 0.0001	Control versus cAMP cAMP versus Aglepristone cAMP versus Mifepristone	*P* < 0.001 *P* < 0.001 *P* < 0.01
*PTGS2*	1 ± 0.00	38.77 ± 14.14	5.89 ± 2.58	6.35 ± 2.20	*P* < 0.0001	Control versus cAMP cAMP versus Aglepristone cAMP versus Mifepristone	*P* < 0.001 *P* < 0.001 *P* < 0.001
*NFKBIA*	1 ± 0.00	0.63 ± 0.22	0.88 ± 0.20	1.17 ± 0.28	*P* < 0.0001	Control versus cAMP cAMP versus Mifepristone Aglepristone versus Mifepristone	*P* < 0.01 *P* < 0.001 *P* < 0.05
*LIFR*	1 ± 0.00	0.31 ± 0.10	0.66 ± 0.20	0.59 ± 0.24	*P* < 0.0001	Control versus cAMP cAMP versus Aglepristone cAMP versus Mifepristone	*P* < 0.001 *P* < 0.001 *P* < 0.01
*CDKN1A*	1 ± 0.00	6.01 ± 1.85	3.78 ± 0.73	3.34 ± 0.64	*P* < 0.0001	Control versus cAMP cAMP versus Aglepristone cAMP versus Mifepristone	*P* < 0.001 *P* < 0.001 *P* < 0.001
*AKAP12*	1 ± 0.00	3.71 ± 1.15	2.66 ± 0.75	2.63 ± 0.40	*P* < 0.0001	Control versus cAMP cAMP versus Aglepristone cAMP versus Mifepristone	*P* < 0.001 *P* < 0.05 *P* < 0.05
*SELP*	1 ± 0.00	4931.65 ± 1458.29	2113.72 ± 476.03	2123.82 ± 726.23	*P* < 0.0001	Control versus cAMP cAMP versus Aglepristone cAMP versus Mifepristone	*P* < 0.001 *P* < 0.001 *P* < 0.001
*ITGA9*	1 ± 0.00	0.25 ± 0.07	0.38 ± 0.09	0.41 ± 0.05	*P* < 0.0001	Control versus cAMP cAMP versus Aglepristone cAMP versus Mifepristone	*P* < 0.001 *P* < 0.01 *P* < 0.001
*LAMA4*	1 ± 0.00	4.73 ± 1.64	3.52 ± 0.95	2.94 ± 0.51	*P* < 0.0001	Control versus cAMP cAMP versus Mifepristone	*P* < 0.001 *P* < 0.01
*CDH1*	1 ± 0.00	0.24 ± 0.07	0.25 ± 0.05	0.25 ± 0.03	*P* < 0.0001	Control versus cAMP	*P* < 0.001
*EDNRB*	1 ± 0.00	57.18 ± 18.05	18.52 ± 9.55	15.42 ± 3.11	*P* < 0.0001	Control versus cAMP cAMP versus Aglepristone cAMP versus Mifepristone	*P* < 0.001 *P* < 0.001 *P* < 0.001
*THBS2*	1 ± 0.00	2.88 ± 0.81	1.52 ± 0.31	1.51 ± 0.39	*P* < 0.0001	Control versus cAMP cAMP versus Aglepristone cAMP versus Mifepristone	*P* < 0.001 *P* < 0.001 *P* < 0.001
*VEGFR1/FLT1*	1 ± 0.00	0.26 ± 0.14	1.93 ± 0.40	1.13 ± 0.55	*P* < 0.0001	Control versus cAMP cAMP versus Aglepristone cAMP versus Mifepristone Aglepristone versus Mifepristone	*P* < 0.001 *P* < 0.001 *P* < 0.001 *P* < 0.001
*ANGPT4*	1 ± 0.00	0.20 ± 0.08	0.46 ± 0.18	0.59 ± 0.31	*P* < 0.0001	Control versus cAMP cAMP versus Aglepristone cAMP versus Mifepristone	*P* < 0.001 *P* < 0.05 *P* < 0.001
*SLC2A1*	1 ± 0.00	3.46 ± 1.10	1.41 ± 0.49	1.15 ± 0.23	*P* < 0.0001	Control versus cAMP cAMP versus Aglepristone cAMP versus Mifepristone	*P* < 0.001 *P* < 0.001 *P* < 0.001
*LDLR*	1 ± 0.00	0.81 ± 0.23	1.14 ± 0.13	1.19 ± 0.17	*P* < 0.0001	cAMP versus Aglepristone cAMP versus Mifepristone	*P* < 0.001 *P* < 0.001
*HIF1α*	1 ± 0.00	0.91 ± 0.33	1.08 ± 0.34	0.84 ± 0.16	*P* = 0.1249	–	

Relative gene expression (RGE), as determined by semi-quantitative real-time TaqMan qPCR is presented as mean and standard deviation (SD). Differences between different treatment groups were evaluated with a non-parametric one-way ANOVA that, when *P* < 0.05, was followed by a Tukey–Kramer multiple-comparisons test.

The treatment of decidualized DUS cells with either antigestagen led to a significant decrease of mRNA availability encoding for *AREG*, *HGF*, *PAPPA2*, *CXCL10*, *PTGS2*, *CDKN1A*, *SELP*, *EDNRB, THBS2* and *SLC2A1* (*P* < 0.001), and of *IL1R1* (*P* < 0.05 for aglepristone and *P* < 0.001 for mifepristone), *TGFR2* (*P* < 0.001 for aglepristone and *P* < 0.01 for mifepristone) and *AKAP12* (*P* < 0.05) (Table 1). Additionally, a significantly decreased expression of *LAMA4* could be observed for mifepristone (*P* < 0.01) but not for aglepristone (*P* > 0.05) (Table 1). Both antigestagens were further associated with the increased expression of *ITGA9*, *LDLR1 (P* < 0.001 for all factors and both antigestagens), *LIFR* (*P* < 0.001 for aglepristone and *P* < 0.01 for mifepristone), and *ANGPT4* (*P* < 0.05 for both antigestagens) (Table 1). Although *VEGFR1* expression was significantly upregulated by both antigestagens when compared with the cAMP group (*P* < 0.001 for both antigestagens), aglepristone induced a significantly higher increased expression when compared with mifepristone (*P* < 0.001). The expression of *NFKBIA* was the highest after mifepristone treatment when compared with the cAMP group (*P* < 0.001), or with aglepristone (*P* < 0.05) (Table 1), which had no significant effects on the availability of *NFKBIA* in decidualized DUS cells (*P* > 0.05, Table 1). Furthermore, the expression of *CHD1* remained unaffected by either antigestagen (*P* > 0.05, Table 1). Finally, no significant differences between different treatment groups could be observed for *HIF1α* or *ANXA2* (*P* = 0.124 and *P* = 0.431 respectively, Table 1).

## Discussion

The crucial role of decidual cells in the maintenance and termination of canine pregnancy makes these cells an important target for investigating embryo-maternal communication in the dog. The particular importance of these cells arises from their sole placental expression of PGR, mediating P4 signaling^12,13^. Therefore, the availability of an in vitro model with DUS cells provides a unique opportunity to gain a deeper insight into possible cell-specific mechanisms underlying canine placental physiology. Building on our previous reports^8,22–24^, the use of transcriptional analysis provided a broader overview of the morphological and functional changes associated with the decidualization of canine stromal cells. Furthermore, following the hypothesized importance of P4/PGR signaling for decidual cell function^23^, deeper insights were obtained into the effects evoked by antigestagens in decidualized DUS cells.

### Decidualization-associated effects

DUS cells were decidualized using the cAMP-mediated approach. Thus, the enrichment of functional terms associated with protein kinase activity, as well as the activation of the canonical pathway PKA signaling (associated with the increased availability of factors involved in kinase activity, e.g., *AKAP12*), were expected. In addition, fitting well with our previous findings^8,23,24^, decidualization markers like *IGF1*, *PTGES* and *PTGS2*, were among the genes with significantly increased expression following cAMP treatment. Among the predicted upstream regulators for this contrast was PGE2. Indeed, the involvement of the prostaglandin family in canine decidualization was described previously^22^, with PGE2 being able to decidualize DUS cells in vitro through its cAMP-associated receptors PTGER2 and -4. Furthermore, although P4 itself does not induce spontaneous decidualization in the dog^8^, it can, at least in part, modulate DUS cell activity by regulating the expression of PGE2 receptors^22^. In humans, where P4 can spontaneously induce decidualization, there is a similar interplay between P4 and PGE2 receptors^25^.

In response to decidualization, several of the identified DEGs were associated with increased cellular proliferation and differentiation, enrichment of functional terms related to mesenchymal-epithelial transition, as well as to modulation of the extracellular matrix. Underlying that, different genes associated with cellular proliferation were among the more strongly upregulated DEGs in cAMP-treated samples, when compared with control DUS, e.g., *AREG*, *PAPPA2* or *HGF*. The significantly increased *AREG* and *HGF* mRNA availability was further confirmed by semi-quantitative PCR. A member of the epidermal growth factor family, *AREG*, was previously associated with embryo implantation and regulation of endometrial function in early pregnancy in the mouse^26^. Furthermore, the expression of AREG, in addition to other members of the epidermal growth factors family, is associated with uterine receptivity and modulation of trophoblast invasion in humans^27^. The upregulation of these growth factors in decidualized DUS cells was accompanied by higher mRNA amounts of *SLC2A1/ GLUT1*. An increased availability of this glucose transporter was previously described in human and murine endometrium during the secretory phase^28,29^, and in human endometrial stromal cells after decidualization^30^, associated with increased energy requirements of the tissues.

Different members of the bone morphogenetic protein (BMP) family were also among the growth factors upregulated after the decidualization of DUS cells. In the murine model, disruption of different BMPs’ function is associated with deficiencies in decidualization and implantation, associated, e.g., with dysregulation of prostaglandin synthesis and Wnt signaling^31–33^. Wnt signaling is implicated in the success of pregnancy establishment in humans and mice, being strongly associated with the mesenchymal-epithelial transition of endometrial stromal cells undergoing decidualization^34,35^. This transition of cellular phenotype is crucial for the formation of the decidua and for uterine receptivity in humans and rodents, involving not only a change in cellular shape, but also the attachment to the basal membrane, differential expression of ECM components and formation of strong cell-to-cell adhesion^34^. In this context, Wnt signaling can trigger several intracellular processes in decidualizing cells, involving, e.g. LIF, BMPs, FOXO1, FGFs, selectins and prostaglandins^35^. Similarly, in our study, Wnt signaling was enriched for DEGs modulated during DUS cell decidualization and was associated with the modulation of Wnt-related factors such as *BMPs*, *LIFR* or *SELP*. Whereas the expression of *LIFR* was downregulated during decidualization of DUS, its ligand LIF was among the upregulated DEGs. An increased expression of *LIF* was also reported in the canine endometrium during placentation^36^. In humans and mice, LIF can regulate decidualization and is associated with implantation and trophoblast invasion^37,38^. Thus, the increased expression of LIF in DUS cells might be associated with endometrial receptivity. Furthermore, SELP belonged to the most strongly upregulated DEGs in decidualized cells. It is involved in several mechanisms, spanning from increasing immune cells infiltrate^39^ to being associated, with other selectins, in the support of the uterine receptivity and implantation process^40,41^. Thus, although still obscure, the involvement of Wnt signaling in the regulation of canine mesenchymal-epithelial transition appears plausible.

In the dog, a mesenchymal-epithelial transition of endometrial stromal cells has been described during the decidualization process^8,9,22,24^. Besides morphological changes, it involves increased expression of epithelial markers (e.g., COL4), ECM components like ECM1, and gap junction components like CX43, while retaining the expression of mesenchymal factors (VIM and α-SMA)^8,22–24^. In the present analysis, functional terms associated with the mesenchymal-epithelial transition were enriched, and represented by factors involved in the modulation of extracellular matrix and cell adhesion, e.g. *LAMA4* (an ECM glycoprotein), as also observed in humans^42^. The modulation of ECM factors in the canine uterus throughout pregnancy was previously observed and was associated with the increased presence of ECM1, TIMP2 and -4, FN1 and LAMA2^43^. The modulation of ECM-related factors might thus be associated with tissue remodeling required for trophoblast invasion and placentation. In this regard, the expression of the adhesion molecule *CDH1* (that encodes e-cadherin) and *ITGA9* was downregulated, together with functional terms associated with integrin signaling being enriched in downregulated DEGs. Integrins play important roles in cell survival and motility, as well as in cell–cell and cell–matrix adhesion^44,45^. Besides their role in modulating trophoblast adhesion, they can also modulate the expression of other adhesion molecules like *CDH1*, downregulated in the present analysis, through their integrin-link kinase (ILK) in different tissues^44,46^. Thus, although still requiring further investigation, the modulation of several ECM factors and adhesion molecules by decidual cells might be important for local tissue remodeling and facilitate trophoblast adhesion and invasion.

Decidualization was further associated with the modulation of angiogenic factors expressed by DUS cells, with several angiogenesis-related functional terms being enriched for the DEGs obtained from the contrast “cAMP versus control”. The decreased expression of *EDNRB* following decidualization was confirmed in real-time PCR analysis. This was accompanied by the increased availability of other endothelin family members observed in the transcriptomics analysis, despite having a lower log2 ratio, such as *EDNRA* and the endothelin converting enzyme 2 (*ECE2*). Endothelins are involved in the regulation of vasodilation/constriction, with the receptor B (encoded by *EDNRB*) inducing vasodilation^47^. Human decidualization is also associated with increased expression of EDNRB^48^, that was further associated with the regulation of trophoblast invasion and cell proliferation during the implantation period^49^. Importantly, endothelins are associated with the onset of preeclampsia in humans, and hypertension linked with placental ischemia in EDNRB-negative mice can be partially reversed by targeting downstream factors from receptor B^50^. Although the pathophysiology is not yet fully understood, preeclampsia is associated with deficient vascularization of the placenta, that leads to maternal high blood pressure and renal disfunction, among other malfunctions^51^. The dog has a less invasive (shallow) endotheliochorial placenta than the hemochorial placenta observed in humans and rodents^9^.Therefore, the physiologically restricted invasion observed in the canine placenta could possibly have some similarities to the shallow invasion observed in placentas from women with preeclampsia, which could suggest the dog as a model animal^52^.

Another factor frequently associated with preeclampsia is the soluble form of the VEGFR1, sFLT1^51,53,54^. VEGF is important for endometrial angiogenesis and vascular remodeling, and fms-like tyrosine kinase (FLT1 or VEGFR1) is one of its well-known receptors^55^. The sFLT is the alternative splice variant and soluble form of the receptor, that acts as an antiangiogenic factor^56^. In our dataset, the expression of the *FLT1* gene was downregulated after decidualization of DUS cells. The decreased production of sFLT1 by primary human endometrial stromal cells after in vitro decidualization^57^ might suggest a similar effect in the dog. However, the differentiation between amounts of the membrane or soluble receptors encoded by this gene could not be performed with the applied methodology, and so still requires confirmation for the dog. In addition, other vasomodulatory factors were differentially expressed after DUS cell decidualization, with *THBS2* presenting increased transcript levels while *ANGPT4* was downregulated. Altogether, the expression pattern of selected candidate vascular factors, as well as the enriched angiogenesis-related functional terms, suggest an association of decidualized cells with vascular remodeling and increased blood flow. This might be further associated with the intimate contact between decidual cells and maternal blood vessels in the canine placental labyrinth^9^. Nevertheless, a broader analysis of other vascularization-associated factors is still required. Furthermore, as the current work employed only DUS cells, further investigations into cellular interactions with other cellular populations in the placenta are still required to fully characterize the vasomodulatory roles of decidual cells in the dog.

In contrast with other selected candidate genes, *PAPPA2* (pappalysin or pregnancy-associated plasma protein A2) transcripts could only be detected in decidualized cells by PCR, and not in control DUS cells. *PAPPA2* is a metalloproteinase that, by cleaving specific insulin growth factor (IGF) binding proteins (IGFBP), releases IGF1, making it available to bind to its membrane receptors^58,59^. The uterine availability of *PAPPA2* was previously described to be decreased in the presence of the embryo during the pre-implantation period, when compared with non-pregnant bitches^60^. Therefore, the increased transcriptional availability of *PAPPA2* following decidualization might be associated with a local increased availability of *IGF1*, a well-described canine decidualization marker^8,22–24^. Although its functional role in the dog still remains to be defined, IGF1 was recently associated with the modulation of cytotoxic activity in decidual natural killer (NK) cells and was shown to promote the survival of human decidual cells^61^. Accordingly, the increased presence of NK cells was recently described in the canine endometrium at the time of implantation, associated with marked local anti-inflammatory signaling^62^. In humans, decidual NK cells are associated with the remodeling of spiral arterioles during the formation of the decidua^63^. Although the exact function of uterine NK cells in the dog still remains to be elucidated, it appears plausible that *IGF1* could further be involved in the modulation of their activity in the canine endometrium.

The immune system plays a crucial role in embryo-maternal communication. In the dog, there is an increased availability of cytokines, including IL1, -6, -8, -10 or CCR7, in the uterus in response to the free-floating embryo^60,62^. Implantation and maintenance of pregnancy, however, appear to be associated with the presence of an anti-inflammatory milieu^62,64^. Here, NFκB was found to be among the predicted upstream regulators in the contrast “cAMP versus control”. In this context, the decidualization-induced downregulation of *NFκBIA* (inhibitor of NFκB) might be associated with the increased immune-related activity of decidual cells. Decidualization also appears to be associated with an increased sensitivity of DUS cells to IL1, concluding from the upregulated presence of *IL1R*. Indeed, increased *IL1β* transcriptional availability was observed during the post-implantation period in canine pregnancy^62^. Furthermore, although *TGFβ* appears to be stably expressed in the placenta throughout canine pregnancy^62,64^, the upregulation of *TGFBR2* suggests an increased sensitivity of decidualized cells to this cytokine. Decidualization was further associated with an increased mRNA availability *CXCL10*, a chemoattractant of T and NK cells^65,66^. In fact, CXCL10 was among the most strongly upregulated DEGs. Conversely, the in vitro decidualization of human endometrial stromal cells leads to a decreased secretion of CXCL10, albeit these effects appear to be associated with the activity of hCG and decreased infiltration of cytotoxic T cells^67^. Although protein and functional analyses are still needed to confirm the nature of the immunomodulatory function of canine decidual cells and to compare these effects with other species, the present study is the first to address their immunomodulatory potential.

### Antigestagen-mediated effects

To obtain deeper insights into PGR-dependent effects in the function of canine decidual cells, decidualized DUS cells were treated with type II antigestagens. In a previous study, aglepristone and mifepristone caused some different effects in decidualized DUS cells, with mifepristone exerting stronger effects than aglepristone on the expression of, e.g., *COL4* and *PGR*^23^. Hence, despite the structural similarity of the two antigestagens, with aglepristone being a derivative of mifepristone and both acting as abortifacients^16^, aglepristone and mifepristone appear to have functional differences. Accordingly, the transcriptional effects exerted by both antigestagens upon decidualized DUS cells differed to a large extent, with only approximately 50% of all identified DEGs being concomitantly affected by both PGR-blockers. These differences were further confirmed for different candidate genes, with the transcriptional availability of *LAMA4* and *NFKBIA* being significantly modulated solely by mifepristone, and significant differences between aglepristone and mifepristone-induced effects on *VEGFR1*/*FLT1* expression. These results highlight that the functional differences between aglepristone and mifepristone are still poorly explored. Yet, the use of both antigestagens in our study substantiated the PGR-mediated nature of the transcriptional effect results observed here, especially with regard to the overlapping DEGs. A high number of genes jointly modulated (up- or downregulated) by both antigestagens overlapped with DEGs presenting opposite expression patterns in the contrast “cAMP versus control”, i.e. their expression was reversed by antigestagens. This is in accordance with observations from the previous report, in which antigestagens decreased, at least in part, the expression of, e.g., decidualization markers (*PTGES*, *PRLR*, *IGF1*)^23^. Here, antigestagens reversed the expression of DEGs associated with cellular proliferation, migration and apoptosis, cellular response to stimulus, cell–cell junction, cell differentiation and regulation of epithelial-mesenchymal transition, cumulatively suggesting broader effects of PGR-blocking in decidualized DUS cell function. This seems to be also associated with the modulation of intracellular cAMP/PKA signaling, as both antigestagens decreased the expression of the PKA-associated factor *AKAP12*, and the PKA pathway signaling was predicted to be deactivated in response to both antigestagens. Accordingly, in our ongoing kinomics analysis, PKA activity was predicted to be increased after decidualization, but decreased by both aglepristone and mifepristone in DUS cells (*own data, unpublished*). In this context, it should be mentioned that, besides genomic signaling, PGR can also induce downstream effects through a non-genomic pathway involving secondary messengers like PKA^68^. Furthermore, P4 can also act through membrane-bound receptors, associated, i.a., with different intracellular kinases^68,69^. Nevertheless, to our knowledge, possible interactions between antigestagens and P4 membrane receptors remains to be defined. The administration of antigestagens to decidualized DUS cells also led to the predicted inactivation of the prostanoid synthesis pathway, linked to *PTGS1, -2* and PGE2 receptor *PTGER4*, that were among the factors downregulated by both antigestagens in the pairwise analysis. The decreased mRNA levels of *PTGS2* were also confirmed in the real time PCR analysis, in addition to the suppressive effects of antigestagens on *PTGES* transcriptional availability shown previously^23^. These observations further highlight the P4/PGR-PGE2 interaction in the decidualization and function of DUS cells^22^.

The enrichment of antiproliferative and proapoptotic functional terms in response to antigestagens was underlined by the decreased expression of factors involved in cellular growth, like *AREG*, *HGF*, *PAPPA2* and *ANXA2*. In mice, mifepristone decreased the expression of *AREG* in the uterus, disrupting uterine receptivity and implantation^26,70^. Furthermore, *CDKN1A*, associated with the differentiation of stromal cells in mice during decidualization^71^, as well as the expression of mediators of glucose and cholesterol transport, i.e., *SLC2A1* and *LDLR*, were downregulated by antigestagens. These apparently adverse effects of antigestagens upon life cycle and viability are supported by the previous findings indicating antigestagen-induced downregulation of the proliferation marker PCNA, occurring concomitantly with increased expression of activated-caspase 3^23^. Furthermore, regarding tissue composition and cell–cell interaction, antigens induced stronger effects on the expression of the tight junction component CX43/GJA1, which was among the DEGs downregulated by both antigestagens in this study, than on ECM1 or COL4 in decidualized DUS cells^23^. Similarly, here, the effects induced by antigestagens upon cell adhesion and tight junctions prevailed over the enrichment of functional terms associated with ECM composition. The proapoptotic events could be, at least in part, related to the disruption of gap junctions as in decidualized human endometrial stromal cells the disruption of *CX43*/*GJA1* activity induced their apoptosis^72^.

In human decidualized stromal cells, disruption of gap junctions was also associated with vaso- and immunomodulatory effects^73^. In accord with these observations, both aglepristone and mifepristone were associated with the modulation of functional terms associated with vascularization, mainly enriched in downregulated DEGs in treated DUS cells (discussed in more detail elsewhere), as well as with immune function, represented by decreased expression of *CXCL10, IL11* and *CXCR4.* Conversely, mifepristone upregulated *NFKBIA*, and both antigestagens increased the expression of *LIFR*. In the dog, parturition, either at term or induced by aglepristone in mid-pregnancy, is associated with increased immune activity in the placenta^64^. The similarities observed in the immune placental milieu in both situations, i.e. during natural and induced parturition imply an immunomodulatory role of P4 in the dog^64^. In fact, immune factors, including *IL1* and *-8*, *CXCR2* and *PTGS2*, were also among the predicted P4-modulated factors^21^, and were also represented by predicted activated signaling pathways in IPA analysis. Thus, the antigestagen-mediated immunomodulatory effects observed here reinforce the hypothesis that decidual cells, through their PGR expression, might be involved in the regulation of placental immune activity in the canine placenta throughout pregnancy.

### Antigestagen effects in vitro versus parturition in vivo

In an attempt to identify decidual cell-associated signaling pathways or factors implicated in the termination of canine pregnancy involving P4/PGR signaling, the present dataset was compared with the placental transcriptional signature during prepartum luteolysis^21^. The enrichment of apoptosis-related functional terms was accompanied by the modulation of factors associated with cellular proliferation and activity, e.g., *SLC2A1*, *ISR2*, *IGF1* and *WNT4*. These factors, in addition to the decidualization-associated factors *PRLR* or *PTGER2*, were previously predicted to be downstream factors for P4^21^. Although still requiring confirmation, part of these effects might be associated with *AKAP12*, which was modulated in all three sets of comparisons (contrasts). This scaffold protein regulates PKA and PKC activity by tethering the kinases to intracellular targets; it is associated with actin skeleton remodeling and cAMP-responsive element binding protein (CREB), regulating this way the cellular proliferation and activity^74,75^. Nevertheless, the role of this anchoring protein still requires further investigation in the dog. In association with tissue composition, several factors involved in extracellular matrix composition and modulation (e.g., *LAMA4* and several MMPs), as well as factors involved in cell adhesion and communication (e.g., *SELP* or *THBS1/-2*), were concomitantly affected in vivo at term and in antigestagen-treated decidualized DUS cells. Furthermore, vasomodulatory factors like VEGF-family members, *ENDRB*, *TGFBR2* and *THBS2* were also concomitantly affected in vitro by antigestagens and during parturition in vivo^21^. All these observations further support the involvement of decidual cell-mediated P4/PGR signaling, possibly in a great part through the cAMP/PKA pathway, in cellular proliferation, cell adhesion, tissue structure and homeostasis in the canine placenta.

## Conclusions

This transcriptional analysis provided a broader overview of the changes induced in the transcriptional signature of canine uterine stromal cells during the decidualization process. Furthermore, new insights into antigestagen-mediated blocking of PGR activity in decidualized cells could be obtained and compared with canine parturition. Our main findings and predicted regulatory events are summarized in Fig. 6D. The cAMP/PKA mediated decidualization, involving, i.a., PGE2, is associated with increased cellular proliferation and differentiation. Concomitantly, the morpho-functional differentiation of DUS cells associated with the mesenchymal-epithelial transition, modulation of ECM composition and increased cellular adhesion, takes place as shown here and is supported by previous findings^8,22,24^. Decidual cells appear to be multidirectionally involved in regulating canine placental function, including local vasomodulatory and immune function. Further, they not only contribute to tissue remodeling, but also appear to regulate endometrial receptivity to the embryo and the species-specific shallow invasion of the trophoblast. The translational potential of these mechanisms to other species, e.g. the aberrant trophoblast invasion observed during human preeclampsia, needs to be emphasized. The hypothesized key role of P4 signaling for decidual cell function is supported by the observed antigestagen-mediated effects in decidualized DUS cells. Antigestagens appear to disturb PKA signaling, implied by overrepresented GOs and functional pathways, as well as to disrupt cellular interactions, possibly leading to pro-apoptotic and antiproliferative events. Further, immunomodulatory effects of antigestagens were observed. Interestingly, several DEGs, as well as functional terms affected by antigestagens, possibly associated with the disruption of feto-maternal communication, coincided with the transcriptional changes observed in the placenta at parturition^21^. The factors identified in the present analysis might represent, at least in part, important decidual cell-derived factors in the maintenance of placental homeostasis and/or active signals related to the initiation of parturition.

## Materials and methods

### Cell culture and in vitro experiments

In all cell culture experiments, the immortalized dog uterine stromal (DUS) cell line, established previously in our laboratory^8^, was used. For culture of cells, including decidualization and antigestagen treatment, the previously described protocols were followed^8,22–24^. Briefly, cells were cultured to at least 80% confluence in 150 cm^2^ cell culture flasks (Corning, New York, NY, USA) using maintenance medium, i.e., DMEM-High Glucose (Bio Concept, Allschwil, Switzerland) supplemented with 10% heat inactivated fetal bovine serum (FBS, Thermo Fisher Scientific AG, Reinach, Switzerland), 100 U/mL penicillin and 100 g/mL streptomycin (PANBiotech, Aidenbach, Germany) and 1% insulin-transferrin-selenium (ITS, Thermo Fisher Scientific AG, Reinach, Switzerland). Cells were then trypsinized, plated at 2 × 10^5^ cells per well in 6-well plates (TPP Techno Plastic Products AG, Trasandingen, Switzerland) and incubated with maintenance medium for 24 h to allow their attachment and recovery. Afterwards, cells were incubated for 72 h with stimulation medium, i.e., maintenance medium in which FBS was replaced with 0.01% bovine serum albumin (BSA; SUB001, Canvax Biotech, Córdoba, Spain). In parallel, for induction of decidualization, stimulation medium was supplemented with 0.5 mM dbcAMP (D0627, Sigma-Aldrich Chemie GmbH, Buchs, Switzerland). Following decidualization, cells were incubated with medium containing either 1 μM mifepristone (Sigma-Aldrich Chemie GmbH, Buchs, Switzerland) or 1 μM aglepristone (Batch No: 2064665, kindly provided by Virbac, Tierarzneimittel GmbH, 23,843 Bad Oldesloe, Germany) for 6 h, with the dosage and time based on our previously described protocol^23^, or with decidualization medium (cAMP control group). The non-treated/non-decidualized cells served as control (C). All experiments were conducted under standard culture conditions, i.e., 37 °C and 5% CO_2_ in air, in a humidified incubator. All experiments were performed five times, using consecutive passages of DUS cells.

### RNA isolation

Total RNA was isolated with TRIzol reagent (Invitrogen, Carlsbad, CA, USA), according to the manufacturer’s instructions. The concentration and purity of RNA was initially assessed with a NanoDrop 2000 Spectrophotometer (Thermo Fisher Scientific AG, Reinach, Switzerland), and RNA integrity numbers (RIN) were assessed using an Agilent 2200 TapeStation System. Samples submitted for sequencing presented RIN ranging between 9.4 and 10.

### Library preparation and RNA sequencing (NGS)

A total of 20 RNA samples (n = 5 for each experimental group, consecutive passages serving as biological replicates) were sequenced using next generation sequencing (NGS) technology, following our previously published workflow^76^. To avoid possible batch effects, all processing steps were done at the same time for all samples. RNA quality and quantity was assessed with a Fragment Analyzer (Agilent, Waldbronn, Germany) to ensure a 260 nm/280 nm ratio between 1.8 and 2.1 and a 28S/18S ratio within 1.5–2. Following this quality control, each RNA sample (100–1000 ng) was processed with the TruSeq Stranded mRNA (Illumina, Inc., City, California, USA) for library preparation. RNA samples were enriched by poly A selection and subjected to reverse transcription. The double stranded cDNA obtained was randomly fragmented, end-repaired with a poly-A tail and ligated to TruSeq adapters containing unique dual indices for multiplexing. Then, fragments having TruSeq adapters were enriched with PCR. The quality and quantity control of the enriched libraries were assessed with the Fragment Analyzer, and samples were normalized to 10 nM in Tris–Cl 10 mM, pH 8.5, with 0.1% Tween 20. Clusters were generated, using 2 nM of the normalized libraries, and samples were sequenced in the Novaseq 6000 (Illumina, Inc.) following the standard protocol. Sequencing was paired end at 2 × 150 bp or single end at 100 bp. The generated raw data was deposited in NCBI’s Gene Expression Omnibus and is accessible through GEO Series accession number GSE213788 (https://www.ncbi.nlm.nih.gov/geo/query/acc.cgi?acc=GSE213788).

### Data analysis

Sequencing results were uploaded to the SUSHI framework^77,78^ for initial analysis. Reads were aligned to the Ensembl canine genome build CanFam3.1 (http://www.ensembl.org/Canis_familiaris/Info/Index) with the mapper Spliced Transcripts Alignment to a Reference (STAR)^79^. The function *featureCounts* from the R package Rsubread^80^ allowed the quantification of gene expression, and detected genes were considered expressed if a minimum average of 10 reads in at least one group of replicates were detected. Then, pairwise comparison (called contrast) between the different groups was performed with the generalized linear model approach from the Bioconductor package DESeq2^81^, and as previously described^76^. The contrasts defined for this pairwise evaluation were “cAMP versus control”, “aglepristone versus cAMP”, and “mifepristone versus cAMP”. The significance of differential expression was evaluated with the Wald test while the Benjamini–Hochberg algorithm was used to calculate the false discovery rate (FDR, adjusted *P* value) for correction of multiple testing. The lists of differentially expressed genes (DEGs) were then filtered by a *P* value and FDR < 0.01 (i.e. < 1%) before functional analysis, and are provided in Supplementary File 1. Overrepresented biological processes and gene ontologies (GOs) for each contrast were obtained with the online tool Enrichr (http://amp.pharm.mssm.edu/Enrichr/^82^) and further confirmed with the Pantherdb online tool (http://pantherdb.org^83^). Next, the identification of enriched biological networks were obtained with the ClueGO application (V2.5.8)^84^ for the Cytoscape software (V3.9.1)^85^. Finally, Ingenuity Pathway Analysis (IPA, Qiagen, Hilden, Germany) software was used to predict the most significantly affected canonical pathways and identify possible upstream regulators. Concomitantly affected DEGs from different contrasts were visualized with Venn diagrams, using the online tool VENNY (https://bioinfogp.cnb.csic.es/tools/venny/; V2.1). Comparison between the present in vitro data and the DEGs obtained in vivo from canine placenta collected at the time of prepartum luteolysis and mid-gestation were further performed using the publicly available dataset GSE126031 (https://www.ncbi.nlm.nih.gov/geo/query/acc.cgi?acc=GSE126031^21^).

### Expression of selected target genes by semi-quantitative real time TaqMan qPCR

To further validate the sequencing data and investigate selected functional pathways, the mRNA availability of 23 selected target genes was evaluated using semiquantitative real-time TaqMan PCR. Commercially available TaqMan systems were ordered from Applied Biosystems by Thermo Fisher (Waltham, MA, USA). If not available, primers and 6-carboxyfluorescein (6-FAM) and 6-carboxytetramethylrhodamine (TAMRA) labelled probes were designed based on published coding sequences and ordered from Microsynth AG (Balgach, Switzerland). Randomly selected PCR products were sequenced with Sanger methodologies (Microsynth AG) to ensure specificity of custom-made systems. Assay efficiency was evaluated to ensure approximately 100% by performing a Ct slope with tenfold serial dilution of cDNA of different samples, as previously described^86^. The detailed information regarding all primers and probes used is listed in Table 2. Reverse transcription and PCR were performed as previously described^86,87^. For each sample, 1.3 μg of total RNA was subjected to DNase treatment with the RQ1 RNase-free DNase kit (Promega, Duebendorf, Switzerland). Next, cDNA was synthesized with the MultiScribe Reverse Transcriptase, using random hexamers as primers (all obtained from Applied Biosystems). Semi-quantitative real time PCR was run in duplicates in 96-well optical plates, using 5 μl of the obtained cDNA per sample with FastStart Universal Probe Master (ROX, Roche diagnostics AG, Basel, Switzerland). Autoclaved water and non-reverse transcribed RNA (RT-minus control) were used as negative controls. The expression of target genes was quantified using the comparative Ct method (ΔΔCt), calibrated to the expression of control cells (except for *PAPPA2*, where the cAMP group was used; discussed elsewhere) for each experiment, and normalized to the expression of reference genes KDM4A, EIF4H, and PTK2, shown to be stably expressed in dog uterus and DUS cells^23,88^. The selection of applied reference genes was further supported by using the online RefFinder tool (https://www.heartcure.com.au/reffinder/?type=reference), that integrates four different algorithms (Delta CT, BestKeeper, Normfinder and Genorm), for checking their stability. The statistical analysis for PCR results was performed using GraphPad 3.06 Software (GraphPad Software, San Diego, CA, USA). A parametric one-way ANOVA was applied and, if *P* value was less than 0.05, was followed by a Tukey–Kramer multiple comparisons post-hoc test. Numerical data is presented as mean ± standard deviation.

**Table 2:**
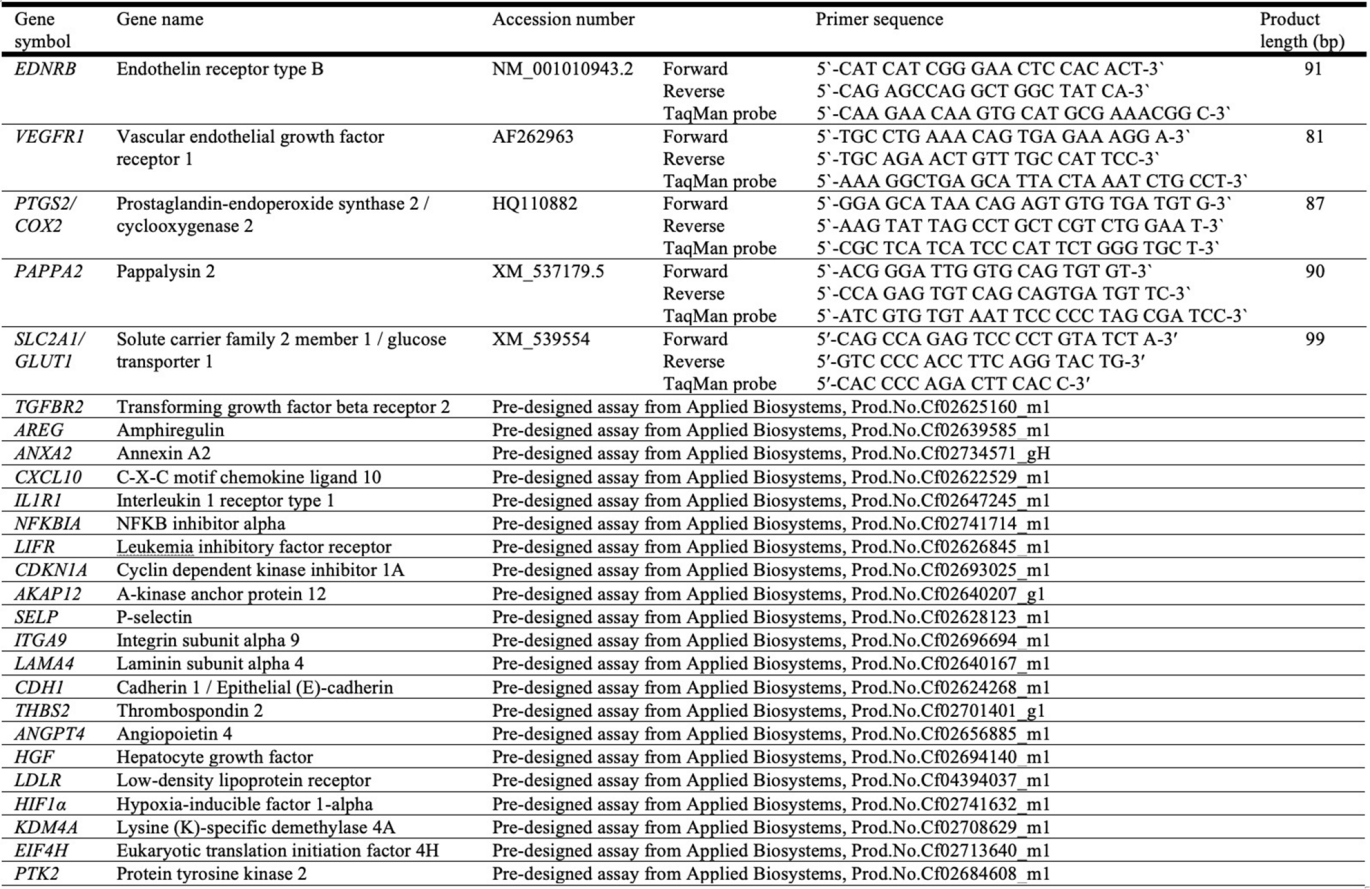
List of symbols, corresponding gene names and TaqMan systems used for semi-quantitative real-time TaqMan PCR.

## Supplementary Information

Supplementary Information 1.

Supplementary Information 2.

Supplementary Information 3.

Supplementary Information 4.

Supplementary Information 5.

## Data Availability

Raw data files (.fastq files) are publicly available in NCBI’s Gene Expression Omnibus with the GEO Series accession number GSE213788. The gene expression data will be available from the corresponding author upon reasonable request.
