# Supplementary figures and images for: Transcriptomic profiling of canine decidualization and effects of antigestagens on decidualized dog uterine stromal cells
Source: Sci Rep. 2022 Dec 19;12:21890. doi: 10.1038/s41598-022-24790-6 (PMC9763427; doi:10.1038/s41598-022-24790-6)

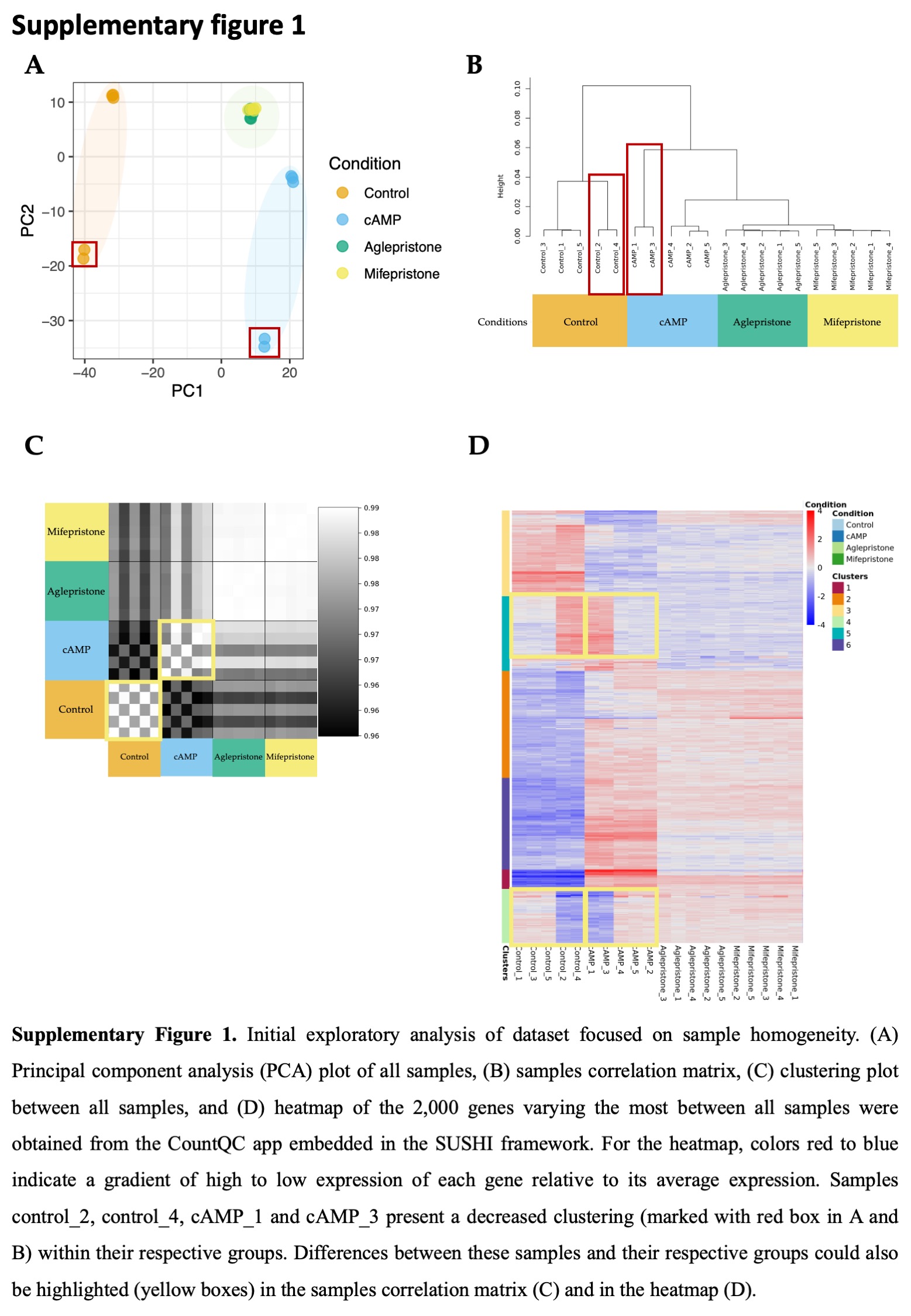

Supplement: Supplementary file 1 — Supplementary Information 1. [file 41598_2022_24790_MOESM1_ESM.jpg]
